# Gene therapy on the move

**DOI:** 10.1002/emmm.201202287

**Published:** 2013-09-17

**Authors:** Kerstin B Kaufmann, Hildegard Büning, Anne Galy, Axel Schambach, Manuel Grez

**Affiliations:** 1 Institute for Biomedical ResearchGeorg-Speyer-Haus, Frankfurt, Germany; 2 Department I of Internal Medicine and Center for Molecular Medicine Cologne (CMMC), University of CologneCologne, Germany; 3 GenethonEvry, France; 4 Institute of Experimental Hematology, Hannover Medical SchoolHannover, Germany; 5 Division of Hematology/Oncology, Children's Hospital Boston, Harvard Medical SchoolBoston, MA, USA

**Keywords:** clinical trials, iPS, monogenic disorders, stem cell therapy, viral vectors

## Abstract

The first gene therapy clinical trials were initiated more than two decades ago. In the early days, gene therapy shared the fate of many experimental medicine approaches and was impeded by the occurrence of severe side effects in a few treated patients. The understanding of the molecular and cellular mechanisms leading to treatment- and/or vector-associated setbacks has resulted in the development of highly sophisticated gene transfer tools with improved safety and therapeutic efficacy. Employing these advanced tools, a series of Phase I/II trials were started in the past few years with excellent clinical results and no side effects reported so far. Moreover, highly efficient gene targeting strategies and site-directed gene editing technologies have been developed and applied clinically. With more than 1900 clinical trials to date, gene therapy has moved from a vision to clinical reality. This review focuses on the application of gene therapy for the correction of inherited diseases, the limitations and drawbacks encountered in some of the early clinical trials and the revival of gene therapy as a powerful treatment option for the correction of monogenic disorders.

## Introduction

Gene therapy involves the use of nucleic acids (DNA or RNA) for the treatment, cure or prevention of human disorders. Depending on the type of disease, this can be achieved either by delivery of a functional, therapeutic gene as a substitute for the defective or missing endogenous counterpart or by reducing the levels of a harmful defective gene product, using various sophisticated tools including naked oligonucleotides, viral and non-viral vectors.

Gene therapy initially focused on orphan diseases with detrimental monogenetic defects, such as primary immunodeficiencies (PID), for which this treatment was considered to be the last, if not the only therapeutic option. The increasing number of successful trials has driven the development of gene therapy approaches to include more widespread applicability, for example, in cancer and chronic or progressive diseases such as heart failure, neurodegenerative or metabolic disorders, including Parkinson's and diabetes (Elsner et al, 2012; Jessup et al, 2011; LeWitt et al, 2011). Although cancer gene therapy accounts now for the majority of clinical trials worldwide (January 2013, http://www.wiley.com//legacy/wileychi/genmed/clinical/), this topic is beyond the scope of this review and readers are referred to recent reviews on this area (Cronin et al, 2012; Lam et al, 2013; Park et al, 2012; Russell et al, 2012; Shen et al, 2012; Wang et al, 2011).

China was the first country to introduce a gene based-drug (Gendicine®), into the market in 2004. Gendicine is an adenovirus-p53 based gene therapeutic approved for the treatment of patients with head and neck squamous cell carcinoma (Wilson, 2005). With more than 10,000 treated patients no overt adverse side effects have been reported for Gendicine®. However, the therapeutic efficacy of this drug is still controversial (Sheridan, 2011; Shi & Zheng, 2009). In Europe, alipogene tiparvovec (also known as Glybera®) was approved for the treatment of familial lipoprotein lipase deficiency (LPLD) at the end of 2012, and thus, was the first commercially available gene therapeutic product in the Western world (Büning, 2013; Miller, 2012; Ylä-Herttuala, 2012). The marketing authorization for Glybera® clearly represents a milestone in the development of gene therapy as an accessible therapeutic option for LPLD patients. The Glybera® example also revealed the multiple layers of complexity that have to be solved before a drug-based product reaches the market. In addition to patent issues, the costs for adequate production of the advanced therapy medicinal product (ATMP) according to good-manufacturing practice (GMP) requirements are enormous. Moreover, costly and extensive pharmacology and toxicology studies have to be conducted in the absence of clearly defined standards, even in cases where very similar vector backbones are used. In addition, the review process and eventual authorization by the respective agencies adds another layer of complexity as exemplified by the hurdles encountered during the review process for Glybera® (as reviewed elsewhere (Bryant et al, 2013)). Thus, there are still multiple issues to be addressed in gene therapy before gene-based products enter routine clinical application to provide safe and affordable therapeutic drugs for otherwise non-treatable overt and chronic diseases.

## *In vivo* and *ex vivo* gene therapy

Multiple gene delivery systems are available, which can either provide transient or stable gene transfer. When the therapeutic effect can be achieved upon expression of a single gene in post-mitotic tissue, non-integrating vector systems are favoured. Indeed, in one of the first *in vivo* clinical trials, an attenuated adenovirus-derived vector was used for the treatment of ornithine transcarbamylase deficiency (OTCD), an inborn disease of urea synthesis (Raper et al, 2002). Vector- and transgene-elicited immunoreactions were initially of concern in the *in vivo* application of vector particles, as documented by the death of one out of the 17 subjects treated in the OTCD trial, which was caused by a massive immune reaction against the capsid of the infused adenoviral vector (Raper et al, 2003). Meanwhile elaborate technologies have been developed not only to shield the viral capsid proteins from recognition by the host immune system, but to successfully implement clinical trials with non-integrating vectors mainly in the area of cancer gene therapy (Cattaneo et al, 2008; Russell et al, 2012).

**Figure 1:**
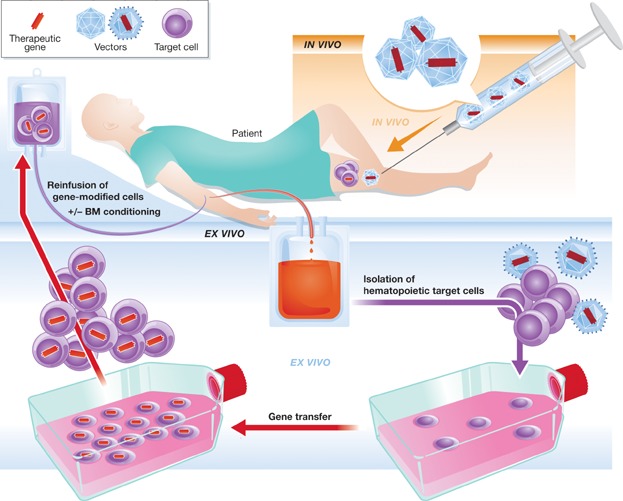
*In vivo* and *ex vivo* gene therapy concepts For the *in vivo* application of gene-based drugs, the therapeutic gene is introduced directly into the body (*e.g*. muscle, liver) of the patient, while for *ex vivo* applications, patient cells are first isolated from the body, genetically modified outside the body and reintroduced into the patient as an autologous transplant (see text for details). BM, bone marrow.

For the correction of monogenic disorders in post-mitotic tissues, adeno-associated virus-derived vectors (AAV) are currently used, as described in detail later. In combination with other characteristics such as low inflammatory activity, they have shown to have an excellent safety profile and are therefore highly attractive tools for *in vivo* gene therapy. Indeed, Glybera® is a recombinant AAV for direct intramuscular injection (Fig 1 and Table 1).

**Table 1:** Overview of clinical trials mentioned in the text

	Target cell/injection	Disease	Transgene	Vector	Refs.
*Ex vivo*	T-lymphocytes	ADA-SCID	*ADA*	Gammaretroviral	Blaese et al (1995)
	HSC	ADA-SCID	*ADA*	Gammaretroviral	Aiuti et al (2002)
		SCID-X1	*IL2Rγ*_*c*_	Lentiviral	Cavazzana-Calvo et al (2000)
		WAS	*WASP*	(±SIN design)	Boztug et al (2010) and Aiuti et al (2013)
		X-CGD	*gp91*^*p*h*ox*^		Ott et al (2006)
	HSC	β-Thalassaemia	*β-Globin*	SIN-lentiviral	Cavazzana-Calvo et al (2010)
	HSC	X-ALD	*ABCD1*	SIN-lentiviral	Cartier et al (2009)
	HSC	MLD	*ARSA*	SIN-lentiviral	Biffi et al (2013)
	HSC	HIV	ZFNs targeting *CCR5* (*knock out*)	Adenoviral	Burnett et al (2012) and Lee et al (2013)
	Hepatocytes	Familial hypercholesterinemia	LDL receptor	Gammaretroviral	Grossman et al (1994)
	T-lymphocytes	B-cell malignancies	Anti-CD19 CAR	SIN lentiviral	Kalos et al (2011), Kochenderfer et al (2010) and Porter et al (2011)
				*SB-transposon*	Swierczek et al (2012)
	Keratinocytes	Epidermolysis bullosa	*laminin 5 β3*	Gammaretroviral	Mavilio et al (2006)
*In vivo*	Intratumoural	Head and neck squamous cell carcinoma	*p53*	Adenovirus (Gendicine®)	Wilson (2005) and Shi & Zheng (2009)
	Intramuscular	LPLD	*LPL*	AAV1 (Glybera®)	Bryant et al (2013) and Kastelein et al (2013)
	Systemic/portal vein	OTCD	OTC	Adenovirus	Raper et al (2002, 2003)
	Subretinal	LCA	RPE65	AAV2	Bainbridge et al, 2008; Hauswirth et al, 2008; Maguire et al, 2008
	Intracerebral (subthal. nucl.)	Parkinson's disease	GAD	AAV2	Kaplitt et al (2007) and LeWitt et al (2011)
	Intracerebral	Canavan disease	ASPA	AAV2	Leone et al (2012)
	Intramuscular, systemic/portal vein	Haemophila B	*FIX*	AAV2	Kay et al (2000)
				AAV2, AAV8	Manno et al (2006)
					Nathwani et al (2011)
	Coronary artery infusion	Heart failure	*SERCA2a*	*AAV*1	Jessup et al (2011)

In contrast, retroviral vectors are preferred for the stable gene transfer into proliferating cells, since they have the capability to integrate into the host cell genome. The current protocols include cell isolation from the patient followed by their genetic modification outside the body and subsequent re-introduction into the patient as an autologous transplant (*ex vivo* gene therapy). This lowers the risk of unwanted off-target effects, such as toxicity due to ectopic expression of the therapeutic gene in off-target organs and excludes germ-line transmission. Furthermore, the therapeutic agent can be administered more robustly since the gene-based drug is not subject to metabolic or renal clearance and is less likely to trigger immune responses. Depending on the protocol, *ex vivo* gene therapy may even allow selection, expansion and quality control of the modified cells before reinfusion, thus further improving safety and efficacy (Fig 1).

Pioneering clinical trials have been performed with mobilized haematopoietic stem cells (HSC) cells, as these cells are easily isolated from the blood after G-CSF mobilization. In addition, procedures to introduce gene-modified HSC into patients have profited from the extensive experience accumulated during 50 years of HSC transplantation (HSCT) (Appelbaum, 2007). In parallel to HSC, mature blood cells have been extensively used for a wide variety of gene therapy purposes resulting in a broad spectrum of applications. Indeed, the first application of gene modified haematopoietic cells into humans was performed at the NCI by Rosenberg et al, who introduced a bacterial gene into tumour infiltrating lymphocytes to track the persistence and localization of the cells after re-infusion into patients with advanced melanoma (Rosenberg et al, 1990). Following this proof of principle, the first gene therapy trial aimed at the correction of an inborn disease was based on the genetic modification of T-lymphocytes for the treatment of adenosine deaminase (ADA) deficiency (Blaese et al, 1995). T-lymphocytes have also been extensively evaluated for autologous adoptive cell transfer providing transient immunotherapy ranging from several weeks to more than a decade (Brentjens et al, 2011; Scholler et al, 2012). For example, a new specificity can be introduced into T cells by delivering an endogenous or synthetic receptor, such as chimeric antigen receptors (CAR), which recognize an antigen of choice on cancer cells and thus, facilitate tumour-cell recognition, ultimately leading to formation of an armada of activated T cells and killing of target cells. This approach has been used successfully in clinical trials, for instance by targeting the B-lymphocyte restricted surface molecule CD19 to treat B-cell leukaemia and lymphoma. In 28 reported cases, this procedure was well tolerated with no therapy related severe side effects and promising clinical outcomes including complete remissions (Kalos et al, 2011; Kochenderfer et al, 2010; Porter et al, 2011). Donor-derived T cells have been widely used to induce a graft-*versus*-leukaemia effect in cases of relapse after allogeneic HSCT. However, serious graft-*versus*-host-disease (GvHD) is frequently observed in treated patients leading to impaired quality of life and reduced survival expectancy, thus limiting the potential of this approach. The introduction of inducible suicide genes, which can be activated upon GvHD development, into the T cells allograft allows for a patient-specific modulation of alloreactivity (Di Stasi et al, 2011; Lupo-Stanghellini et al, 2010; Vago et al, 2012). These approaches have been extensively reviewed elsewhere and will not be discussed further in this review (Bonini & Parmiani, 2012; Brenner, 2012; June et al, 2009; Kalos, 2012; Kershaw et al, 2013).

**Glossary**

**Insertional transformation**

Vector-induced dysregulation of gene expression at the site of integration leading to cell immortalization and eventually to tumourigenesis.

**Self-inactivating (SIN)**

Deletions in the U3 region of the 3′ long-terminal repeats (LTR) of a retroviral vector results in a transcriptionally inactive 5′LTR upon reverse transcription reducing the transactivation potential of the vector. The lack of promoter activity is compensated by an internal promoter of choice.

**Engraftment**

Incorporation of graft cells into the host, *e.g*. transplanted donor haematopoietic stem cells engraft in the bone marrow of the recipient.

**Suicide gene**

A gene that induces apoptosis upon activation by a well-defined stimulus.

**Transgene**

Exogenous genes that are delivered by a vector *in trans* are also referred to as transgenes.

**Cross-correction**

The therapeutic gene product is produced by another cell entity than the actual cell type affected by the genetic defect.

**Primary immunodeficiency (PID)**

Inherited disorders manifesting in a compromised immune system.

**G-CSF mobilization**

Injection of the cytokine granulocyte-colony stimulating factor (G-CSF) induces haematopoietic stem cells to translocate from the bone marrow into the blood, a process called mobilization, and thus can be isolated by collecting peripheral blood.

**Designer nucleases**

Artificial restriction enzymes designed to specifically target a locus of interest, *e.g*. to trigger gene correction or gene disruption via induction of cellular DNA repair mechanisms.

**Induced pluripotent stem cells (iPSC)**

Cells derived from non-pluripotent somatic cells that have been reprogrammed to a pluripotent state upon exposure to certain reprogramming agents. They thereby gain the capacity to differentiate into various tissue types similar to the natural pluripotent embryonic stem cells.

Gene therapeutic approaches for skin diseases also offer a promising and easily accessible cell source for topical *in vivo* application (Roos et al, 2009) as well as for *ex vivo* modification as assessed for instance for epidermolysis bullosa, an inherited skin disorder of connective tissue (Mavilio et al, 2006). Following extremely invasive protocols, hepatocytes are also amenable to *ex vivo* gene therapy as they can be isolated from liver, cultured *ex vivo* and after genetic modification reintroduced into the patient via the hepatic portal vein. Indeed, one of the earliest gene therapy trials was conducted by Grossman et al, in 1992 to treat a patient with familial hypercholesterolemia by genetic modification *ex vivo* of cultured hepatocytes (Grossman et al, 1994). With the development of highly efficient vectors this approach has been largely replaced by the direct injection of the therapeutic vector into the portal vein, as discussed later. However, genetic modification of HSC remains the major focus of gene therapy trials for inherited disorders due to the undeniable achievements in the past, not only in terms of experience, efficacy, long-term follow-up and accessibility, but also the rapid translation into clinical Phase I/II trials (Table 1).

## Primary immunodeficiencies in the focus of gene therapy for monogenic disorders

PID comprises a group of rare, inherited disorders of the immune system caused by defects in the development and/or functions of the various cells of the immune system leading to impaired adaptive and/or innate responses, predisposing patients to infections, allergy, autoimmunity and cancer. Depending on the specific causative genetic defect (>190), the phenotypes of PIDs are generally diverse, often overt and result in a highly reduced life expectancy (Casanova et al, 2008; Fischer, 2003; Gathmann et al, 2012; Notarangelo, 2010). Transplantation of HSC from allogeneic HLA-compatible donors is the treatment of choice for patients with PID, resulting in long-term survival of >90% of patients and effective immune reconstitution. For all others however, transplantations from mismatched donors are still associated with a high morbidity and mortality due to autoimmune and inflammatory manifestations, persistent infections, serious GvHD reactions and graft rejection (Honig et al, 2006; Mazzolari et al, 2007; Neven et al, 2009; Railey et al, 2009; Titman et al, 2008). Therefore, genetic modification of the patient's own HSCs has been considered as an attractive therapeutic option for patients lacking compatible HSC donors. Despite their rare overall prevalence (ranging between 1 and 5 in 100,000 inhabitants within Europe) (Gathmann et al, 2012), monogenetic PIDs have several attributes, which made them highly attractive for gene therapy: they require the *ex vivo* delivery of just one single gene into the HSC, for some PIDs there is a natural *in vivo* selection for gene corrected cells and there are well-established protocols for HSC isolation and transplantation (Aiuti et al, 2012; Appelbaum, 2007).

**Figure 2:**
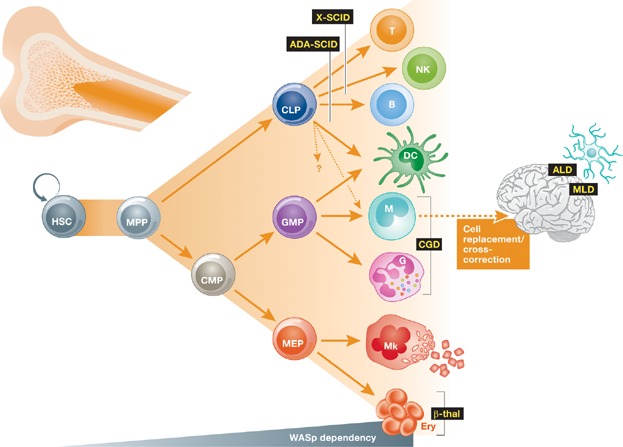
Haematopoiesis and main diseases in focus of *ex vivo* HSC gene therapy The haematopoietic stem cell (HSC) has the ability to give rise to all terminally differentiated haematopoietic effector cells by passing through various intermediate precursor stages. Lineage fate is determined mainly by cytokine profiles which drive development from multipotent progenitors (MPP) to either oligopotent committed lymphoid or myeloid progenitors (CLP and CMP, respectively). CLP eventually provide mature B- and T-lymphocytes, natural killer (NK) cells and dendritic cells (DC). DC can also descend from the myeloid lineage. CMP give rise to megakaryocyte–erythrocyte progenitors (MEP) and granulocyte–monocyte-progenitor (GMP) eventually resulting in either erythrocytes (Ery) and platelet-producing megakaryocytes (Mk) or monocytes (M) and the different entities of granulocytes (G), respectively (adapted from Doulatov et al, 2012). Primary immunodeficiencies (PID) discussed in the text (black) can manifest at several of these stages as indicated, resulting in defects affecting only certain cell types or in complete absence of an entire lineage branch. Gene-transfer in HSC also offers cell replacement or cross-correction of storage diseases of the brain, *e.g*. due to invading monocytes differentiating into microglia.

A genetic defect can affect the haematopoietic system at various stages of haematopoiesis leading to a PID (Fig 2). In some cases disruption in the early stages of lineage commitment leads to a total lack of cell subsets further downstream, as is the case in severe combined immunodeficiency (SCID). This disorder can be subdivided according to the underlying genetic aberration. Currently, gene therapy approaches have mainly focused on two of the most common types of SCID: the autosomal recessive inherited enzymatic defect of the ubiquitously expressed adenosine deaminase (ADA-SCID) that plays role in the purine salvage pathway, and the dysfunction in interleukin-2 (IL-2) signalling due to mutations in the X-chromosomal encoded common gamma chain of the IL-2 receptor (SCID-X1; Aiuti et al, 2009; Candotti et al, 2012; Cavazzana-Calvo et al, 2012; Fischer et al, 2013; Gaspar, 2012; Hacein-Bey-Abina et al, 2002). Phenotypically, patients suffering from these PIDs either lack the lymphocytic compartment including NK cells or their lymphocytes have impaired function (Fig 2). These patients are transplanted soon after birth, if a matched related donor is available. Otherwise, their life expectancy reaches barely beyond infancy (Gathmann et al, 2009). Other PIDs manifest further downstream in the haematopoietic pedigree. In patients with Wiskott–Aldrich syndrome (WAS), thrombocyte and immune cell (but also all other mature blood cells) functionalities are impaired due to a defect in a haematopoietic protein (WASp) responsible for linking receptor signalling to organization of the actin cytoskeleton (Notarangelo et al, 2008). Chronic granulomatous disease (CGD) belongs to the inherited myeloid disorders with no known deficit in haematopoietic cell numbers. The CGD phenotype is characterized by the inability of mature phagocytes to kill ingested microorganisms and eventually manifests as severe and life-threatening granuloma and abscess formation accompanied by hyper-inflammation. The underlying genetic mutations are manifold and the affected genes encode for different subunits of the phagocyte NADPH oxidase complex (gp91^phox^, p22^phox^, p47^phox^, p67^phox^, p40^phox^; Roos, 1994; Segal et al, 1992). In most cases (∼70%), the *cytochrome b(558)* gene (*CYBB*), which is located on the X-chromosome (X-CGD) and encodes for the catalytic subunit gp91^phox^, is affected (van den Berg et al, 2009). Therefore, delivering gp91^phox^ as a transgene is a reasonable approach to treat most of these patients.

## The early years of gene therapy

The very first approved clinical trial for gene therapy for PIDs was initiated in 1990 and addressed ADA-SCID. In this initial study, two children suffering from ADA deficiency were treated repeatedly by autologous transplantation of T-lymphocytes that had been modified *ex vivo* with a functional copy of the ADA cDNA by gammaretroviral vector-mediated gene transfer. Both patients responded positively to the treatment as measured by normalizing T lymphocyte counts in the blood as well as by an increase in ADA enzyme activity in one patient (Blaese et al, 1995). However, the phenotype was not entirely reversed as both patients remained on enzyme replacement therapy (polyethylene glycol conjugated bovine ADA, PEG-ADA), thereby masking the natural *in vivo* selective advantage of detoxification by gene modified cells. Nonetheless, this important study presented the proof-of-concept for the feasibility to treat a genetic disorder by gene therapy without major side effects. Due to low efficiency of transduction and lack of sustained engraftment, following studies used improved gene transfer protocols, slightly altered gammaretroviral vectors and CD34^+^ HSCs as target cells. In addition, a non-myeloablative conditioning regimen was implemented to enhance engraftment of gene-transduced cells (Aiuti et al, 2002; Carbonaro et al, 2012; Gaspar, 2012). To date, more than 40 patients with ADA-deficiency have been treated by gene therapy at different centres in Italy, the UK and the US with impressive success when compared to mismatched allogeneic HSCT as all 40 patients are still alive with excellent reconstitution of immune and metabolic functions. Moreover, the vast majority of patients (*n* = 29) has become independent of PEG-ADA replacement therapy (Aiuti et al, 2009, 2002; Candotti et al, 2012; Gaspar et al, 2011). In a few cases, however, enzyme-replacement had to be reinitiated due to low engraftment and/or low peripheral T-cell counts caused by deficient thymic support. Gene therapy has been also highly successful in the absence of myelosuppressive conditioning in SCID-X1 infants according to functional T-cell reconstitution. Eighteen out of 20 treated SCID-X1 children are alive with full reconstitution of T-cell immune functions, revealing a superior success rate (10% mortality rate) than conventional allogeneic HSCT (25% mortality rate) (Cavazzana-Calvo et al, 2000; Fischer et al, 2011; Gaspar et al, 2011; Hacein-Bey-Abina et al, 2002; Sheridan, 2011; Zhang et al, 2013). This compelling success was favoured by a natural selective advantage for gene-corrected cells, as in both types of SCID, patients are devoid of either all or some lymphocytic lineages offering empty niches for transplanted cells to engraft. Despite this selective advantage gene therapy for older SCID-X1 patients has been less successful, most likely reflecting the loss of thymus regulated T-cell maturation after puberty and emphasizes that the age of the patients at the time of treatment is crucial in some disease contexts. Recent data, however, suggests that non-myeloablative conditioning may improve not only T-cell reconstitution but also B- and NK-cell recovery in older SCID-X1 patients after gene therapy, although the reported follow-up in this patient was too short (3 months as of May 2013) to allow for any conclusive statements (DeRavin et al, 2013).

The resulting enthusiasm in the field, however, was dampened by the occurrence of acute T-cell lymphoblastic leukaemia (T-ALL) in five SCID-X1 patients 2–5.5 years after gene therapy. Four out of these five patients are in remission after chemotherapy and in good condition with detectable gene marking in peripheral blood cells (Cavazzana-Calvo et al, 2012). Initiation of transformation was traced back to insertional activation of the proto-oncogene *LMO2* (LIM domain only 2), a transcriptional cofactor, which in addition to its role in HSC development, promotes self-renewal of committed T cells when overexpressed thereby facilitating the acquisition of additional mutations (McCormack et al, 2010). Indeed, in four out of the five cases of T-ALL, additional leukaemia promoting mutations unrelated to the vector integration event were described (Cavazzana-Calvo et al, 2000; Hacein-Bey-Abina et al, 2003; Howe et al, 2008).

The first three clinical gene therapy trials for CGD were initiated in the late 1990s with limited success as compared to the aforementioned trials addressing ADA- or SCID-X1 (Goebel & Dinauer, 2003; Malech, 1999; Malech et al, 1997). The major difference was observed in the absence of engrafted gene-modified cells. In the first CGD patients (*n* = 12) treated, <1% of circulating peripheral blood cells were transgene-positive a few months after gene therapy, while in gene therapy trials addressing other PIDs full reconstitution of the T-cell compartment was observed in some cases with significant (0.1–16%) gene marking in the myeloid compartment (Aiuti et al, 2007; Cavazzana-Calvo et al, 2005), the target compartment in CGD. Although the protocols were comparable in terms of gene delivery, culture and transduction conditions, gene-modified cells of CGD patients are not known to have survival and proliferative advantages over non-transduced cells, as is the case in SCID-X1 and ADA-SCID, imposing the necessity of (partial) myeloablation previous to the reinfusion of gene modified cells for CGD. Subsequent gene therapy trials addressing X-CGD used mild conditioning regimes as exemplified in a 2004 trial initiated in Frankfurt (Ott et al, 2006). Despite partial conditioning with low myeloablative regimens, long-term engraftment of gene corrected cells has failed in 14 patients treated worldwide to date, suggesting inherent disease-related defects in the stem cell pool (Grez et al, 2011). Nonetheless, all patients showed clear signs of improvement in their clinical conditions early after treatment as documented by the elimination of recurrent, drug resistant infections and reconstitution of superoxide production at therapeutic levels. However, in four patients (two adults in Frankfurt and two children in Zurich) a clonal outgrowth of gene-modified cells was observed 5–15 months after gene therapy. This resulted in the development of a myelodysplastic syndrome (MDS), a pre-leukaemic condition, together with monosomy 7 in three out of the four patients. Clonal dominance was caused by insertional activation of two cell growth promoting genes, namely *MDS-EVI1* and *PRDM16* (Ott et al, 2006; Stein et al, 2010; and J. Reichenbach, Zurich, personal communication). While both children were rescued by allogeneic HSCT and are currently disease-free, both adults succumbed to their underlying disease and leukaemia development.

For the treatment of WAS, gene therapy trials were initiated in Germany in 2007. Excellent reconstitution of WAS protein (WASp) expression was detected in multiple haematopoietic lineages with a clear selective advantage for gene corrected lymphocytes. Resolution of bleeding and eczema correlated with WASp expression concomitant with recovery from autoimmunity (Boztug et al, 2010). Similarly, to the X-CGD gene therapy trial, the WAS trial was initially considered a shining example for successful gene therapy, until leukaemia developed. The first case of T-ALL, again triggered by insertional activation of *LMO2*, was reported in 2010 (Persons & Baum, 2011). Additional leukaemia cases were recently reported (Aiuti et al, 2012; Mukherjee & Thrasher, 2013).

Thus, the need to establish protocols considering the specific disease context was emphasized by the different outcomes observed among the distinct PIDs. Disease-related predisposition for transformation is highlighted by retroviral integrations in the same hotspot (*LMO2*) in trials addressing SCID-X1, WAS and ADA, with >30% of patients developing T-cell leukaemia in SCID-X1 and WAS, compared to ADA-SCID, in which no signs of transformation have been observed after more than 14 years of follow-up (as reviewed in (Cavazza et al, 2013; Fischer et al, 2011)). In line with this, the patient's age, the dose of modified cells and the number of integrated vector copies as well as the therapeutic transgene and its regulation might require individual adjustments. For example, the engraftment failure in CGD patients may require regulated gene expression of the gp91^phox^ protein, as inappropriate expression may induce ROS production in HSC with enhanced differentiation and loss of stemness (Ito et al, 2004, 2006; Juntilla et al, 2010).

In addition, the vector-dependent leukaemia cases emphasized the need for enhanced vector safety, and the development of paradigmatic *in vitro* and *in vivo* assays to prospectively evaluate the safety profile of integrating vectors (Corrigan-Curay et al, 2012; Modlich et al, 2006, 2009; Montini et al, 2009; Schambach et al, 2013). In contrast to conventional allogeneic HSCT, monitoring chimerism and clonal outgrowth of the transplanted gene-modified cells can be accomplished by sequencing and tracing integration sites, thus allowing for an estimation of the abundance of unique clones contributing to gene-marked haematopoiesis (Arens et al, 2012; Brugman et al, 2013). Currently, identification of the clonal repertoire and monitoring of gene-marked cells is simplified, accelerated and rendered more sensitive by next generation sequencing methods allowing early detection of clonal dominance making an early intervention possible before the development of side effects. The understanding of the molecular and cellular basis of clonal imbalance has led to improvements especially in vector design and several clinical trials evaluating these improved vectors have been opened recently or are under way as discussed below in more detail.

## Integrating vectors, risk of insertional transformation and improved vector design

The severe adverse events observed in the early gene therapy trials using gammaretroviral vectors prompted extensive studies on the process of retroviral integration in human cell lines and primary human HSCs (CD34^+^) (Cattoglio et al, 2007; Deichmann et al, 2011; Derse et al, 2007; Mitchell et al, 2004). These studies revealed that gammaretroviral vectors tend to integrate into close proximity to gene regulatory regions (promoters, enhancers, locus control regions) implying a high risk of transcriptional dysregulation, especially since the vector configurations used in these early trials contained an intact 5′ long terminal repeat (LTR), including strong enhancer and promoter elements, which were initially intended to increase therapeutic efficacy. Moreover, the discovery of hot spot regions for retroviral integration augmented the probability of dysregulation of gene expression. This was indeed the case in the SCID-X1 and X-CGD trials, in which a strong increase in either *LMO2* or *EVI1* expression was observed due to insertional activation of these genes at their genomic loci leading to clonal dominance and leukaemogenesis (Hacein-Bey-Abina et al, 2003; Ott et al, 2006). Indeed the genomic loci for *MDS-EVI1* and *LMO2* are currently known to be integration hot-spots for gammaretroviral vectors in murine and human HSCs (Cattoglio et al, 2010; Kustikova et al, 2005). These and further observations led to the development of the self-inactivating (SIN) retroviral vector design with deletions in the U3 region of the 5′LTR resulting in a transcriptionally inactive LTR. The lack of promoter activity is compensated by an internal heterologous promoter driving the transgene expression (as reviewed in (Maetzig et al, 2011; Schambach et al, 2013)). Although the SIN configuration is not known to alter the integration profile of gammaretroviral vectors, the genotoxicity of vectors containing internal cellular or tissue specific promoters is strongly reduced as measured by the potential of these vectors to induce transformation in an *in vitro* immortalization assay (Modlich et al, 2006). Indeed, expression driven by mammalian promoters conferring more physiological levels of expression revealed reduced incidence or even absence of proto-oncogene activation (Zychlinski et al, 2008).

In contrast to gammaretroviral vectors, lentiviral vector insertion sites are rather underrepresented in regulatory regions but revealed a preference for integration into the body of genes. This lowers, but does not completely alleviate, the risk of genotoxicity according to studies addressing the oncogenic potential of these vectors either *in vitro* or *in vivo* (Modlich et al, 2009; Montini et al, 2006, 2009). The common consensus drawn by these studies is that viral vector integration is an active process catalysed by the tethering of the viral preintegration complex to open chromatin regions in the host cell genome as characterized by DNaseI hypersensitive sites and epigenetics marks (Cattoglio et al, 2010; Deichmann et al, 2011; Felice et al, 2009). For example, the host-cell encoded LEDGF/p75 binds to the lentiviral integrase to direct integration to active transcription units. Lentiviral vector integration can be retargeted to heterochromatin regions in the genome by fusing the C-terminal integrase binding domain of LEDGF to the heterochromatin-binding protein 1β (CBX1; Gijsbers et al, 2010). These studies also demonstrated that other retroviral vectors and gene delivery systems such as transposons possess an almost neutral integration profile *ab initio*, that could be considered to be favourable in terms of genotoxicity. Consequently, foamy virus and more recently alpharetrovirus derived vectors have been evaluated in preclinical settings for PIDs since they revealed the least biased integration preferences (Chatziandreou et al, 2011; Derse et al, 2007; Kaufmann et al, 2012; Suerth et al, 2012). For the same reasons, DNA transposon-based vectors, like *Sleeping Beauty* and *piggyBac*, have received considerable attention in the past and are currently under evaluation for gene replacement therapy in a series of applications including HSC, mesenchymal stem cells and myoblasts, among others. Indeed the *Sleeping Beauty* transposon vector system was used to introduce CD19-specific CARs into T cells for the treatment of B-cell malignancies in a Phase I/II clinical trial initiated in 2012 (for a comprehensive review on the *Sleeping Beauty* gene transfer system see (Aronovich et al, 2011; Di Matteo et al, 2012; Hackett et al, 2013; Swierczek et al, 2012)).

In addition to integration, the particular therapeutic gene delivered, the extent of engraftment and the underlying disease might also influence the susceptibility to cellular transformation (Cavazza et al, 2013; Kustikova et al, 2009b). Whether these mentioned possible scenarios ultimately result in the development of an oncogenic process depends strongly on the cell type affected (Kustikova et al, 2009a; Newrzela et al, 2008). Highly proliferative cells, such as progenitor cells, are more prone to transformation by aberrant gene expression of proto-oncogenes or tumour suppressor genes than terminally differentiated cells.

Taken together, vector–chromatin interaction and its consequences have become more predictable, but vector-induced leukaemogenesis remains an unpredictable factor in gene therapy due to its multifactorial nature as described above. However, potential adverse effects have to be balanced against the clinical benefit expected for the individual patient, taking into consideration the clinical complications associated with alternative treatment options, *i.e*. allogeneic HSCT from a mismatched donor. Indeed, the success and feasibility of gene therapy is undeniable considering that the majority of the more than 60 patients treated for ADA-SCID and SCID-X1 within the last two decades experienced a clear clinical benefit. Despite the occurrence of leukaemia in some of these patients the overall success rate of gene therapy outperforms the results obtained after allogeneic HSCT with HLA-mismatched donors.

## The new era of gene therapy

**Figure 3:**
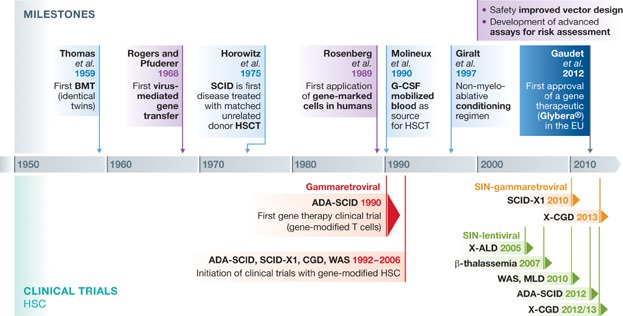
HSC gene therapy timeline History of gene therapy and the milestones that contributed to the implementation of gene therapy for monogeneic disorders using haematopoietic cells (adapted from Appelbaum, 2007; Wirth et al, 2013). Milestones in HSCT are highlighted in light blue whereas major contributions in the field of gene transfer are coloured violet. Although no haematological disorder can be treated with Glybera® (dark blue), its market approval is a milestone for the entire field of gene therapy. BMT, bone marrow transplantation; disease abbreviations as in the text.

The concept of the SIN configuration greatly improved the safety profile of integrating vectors. Not surprisingly, this configuration in combination with physiological or tissue restricted internal promoters has already entered the clinical arena with SIN vector-based trials (Fig 3). Indeed a SIN gammaretroviral vector harbouring the elongation factor short (EFS) promoter driving the expression of ILR2G is currently being evaluated in a multicentre clinical trial for SCID-X1. To date, eight patients have been enrolled in a multinational trial in France, the UK and the US. Although the follow up is relatively short, the initial observations are promising according to kinetics of T lymphocyte reconstitution with partial restoration of humoral immunity mimicking the results seen in the previous trial with LTR-driven vectors but without any sign of leukaemogenesis (Mukherjee & Thrasher, 2013). Similarly, a SIN-gammaretroviral vector was recently approved in Germany for the treatment of CGD. In this case, the vector contains a short myeloid-specific promoter derived from the human c-FES gene controlling the expression of a codon-optimized gp91^phox^ cDNA (Loew et al, 2010; Moreno-Carranza et al, 2009). However, SIN-lentiviral vectors are currently the preferred tool for transferring genes into HSCs (Fig 3), since they possess certain advantageous attributes compared to the gammaretroviral vectors used in the early gene therapy trials (as reviewed in (Naldini, 2011)). Importantly, the preintegration complex of lentiviruses is actively translocated into the nucleus and thereby facilitates efficient transduction of a variety of non-dividing cells. In contrast, other retroviruses such as gammaretroviruses depend on dissolution of the nuclear membrane during mitosis for delivering their cargo into the target cell nucleus. Consequently, efficient transduction of HSCs can be achieved with SIN-lentiviral vectors after a shorter incubation time *in vitro*, preserving to some extent the physiological nature of HSCs and their engraftment potential. Moreover, lentiviral vectors can be easily pseudotyped with envelopes containing vesicular stomatitis virus glycoproteins (VSVg) providing a broad tropism and enabling effective transduction of target cells such as CD34^+^ HSC. The VSVg envelope enables robust manufacturing and purification protocols, which contribute to a superior pharmaceutical quality of these vectors (Merten et al, 2011).

Phase I/II clinical trials with SIN-lentiviral vectors have been initiated for several PIDs (WAS, ADA-SCID, CGD) as well as for non-PID defects amenable to treatment by gene modified HSCs, including X-linked adrenoleukodystrophy (X-ALD), metachromatic leukodystrophy (MLD) and β-thalassaemia.

The most advanced of these studies are the WAS Phase I/II studies ongoing in the UK, France, Italy and the US with a total of 10 patients treated (A. Galy, Evry, personal communication; Aiuti et al, 2013; Hacein-Bey-Abina et al, 2013). In this case, a SIN-lentiviral vector containing a 1.6 kb stretch of the WAS gene's upstream regulatory region was used to drive WAS transgene expression (Aiuti et al, 2013; Charrier et al, 2006; Marangoni et al, 2009; Rivat et al, 2012; Scaramuzza et al, 2013). Unlike the gammaretroviral vector used in the first WAS gene therapy trial, this lentiviral vector provides physiological regulation of the WAS transgene expression in haematopoietic cells. Patients treated with autologous HSC transduced with the WAS lentiviral vector show restoration of WAS protein expression in multiple lineages of leukocytes which led to increased platelet counts, enhanced immune functions and amelioration of the clinical manifestation of the disease (Aiuti et al, 2013; Hacein-Bey-Abina et al, 2013). Similarly, a Phase I/II trial with a SIN-lentiviral vector for the treatment of ADA-SCID is ongoing in the UK and in the US (NCT01380990). Four patients have thus far been treated with a vector containing the EF1α promoter driving the expression of the ADA cDNA. With less than a year of follow-up, no conclusive statements can be made at this time point (Gaspar et al, 2013; Zhang et al, 2013).

For X-CGD, a Phase I/II clinical study using a SIN-lentiviral vector was recently approved in the UK and is currently under regulatory review in Germany and Switzerland. As the functional defect in CGD affects terminally differentiated myeloid cells, attempts were made to target gene expression to this cell population. Promoters derived from endogenous sequences of predominantly myeloid expressed genes, *e.g. MRP8 c-FES* or hsa-miR-223, showed specificity *in vitro* as well as *in vivo*. However, their activity *in vivo* was rather weak (Brendel et al, 2011, 2013; Heydemann et al, 2000). Therefore, several groups addressed this challenge by fusing promoter elements of myeloid restricted genes with other regulatory sequences, *e.g*. the strong viral CMV promoter, regulatory sequences of another myeloid expressed gene (Cathepsin G) or a ubiquitously acting chromatin opening element (UCOE), respectively (Barde et al, 2011; Brendel et al, 2011; Santilli et al, 2011). For X-CGD, the CathepsinG/c-*FES* promoter combination was selected from a series of combinations tested as the most efficient myeloid-specific promoter to drive gp91^phox^ expression in terminally differentiated myeloid cells. Indeed, pre-clinical trials showed excellent tissue restricted expression with low or undetectable expression in stem and progenitor cells while rescuing superoxide production in granulocytes to wild type levels at low vector copy numbers (Santilli et al, 2011). Targeting gene expression to terminally differentiated myeloid cells can be further enhanced by incorporating microRNA (miR) target sequences into the vector backbone. In this case, residual transcripts arising from the myeloid promoters in off-target cells, like HSCs or haematopoietic progenitors, are degraded by miRs differentially expressed in HSCs and progenitor cells, but not in terminally differentiated myeloid cells, as is the case for miR-126 (Lechman et al, 2012). Indeed, the incorporation of two target sites for miR-126 in a lentiviral backbone led to a tight control of gp91^phox^ expression in transduced haematopoietic cells (A. Aiuti, Milan, personal communication).

The genetic modification of HSC also offers the opportunity for treatment of other monogenic disease entities besides PIDs, which are also curable by HLA-matched allogeneic HSC transplantation. This is the case for inborn errors of metabolism such as mucopolysaccharide disorders or lysosomal storage disorders. Similarly, the leukodystrophies, a group of inherited diseases characterized by defects in myelin sheath formation and/or maintenance within the brain, spinal cord and often also the peripheral nerves, can be treated by allogeneic HSC transplantation depending on the stage of the disease and patient age. The mechanisms of HSC-mediated disease correction are based on the replacement of CNS microglia by the progeny of the transplanted haematopoietic cells and/or by a mechanism called ‘cross-correction’, in which monocyte-derived cells secrete a therapeutic enzyme which is then absorbed by enzyme-deficient cells in the CNS (mainly oligodendrocytes and neurons), thereby preventing the neurogenerative manifestation or progression of these disorders (Byrne et al, 2012). Accordingly, gene-modified autologous HSCs may offer a unique opportunity for the treatment of metabolic disorders. This was demonstrated in the first clinical trial using a SIN-lentiviral vector for the correction of X-ALD (Cartier et al, 2009). X-ALD is a severe cerebral demyelinating disease with a strong and progressive neurological phenotype due to a genetic defect in the *ABCD1* gene encoding for the ALD protein, an ATP-binding cassette transporter. The progressive and irreversible nature of this disease warrants intervention as early as possible to allow arrest of demyelination. This was achieved in two treated patients 14–16 months post-transplantation of gene-modified autologous HSC transduced with an *ABCD1* expressing lentiviral vector. Since then the demyelination process has not progressed (overall follow-up 4 years). Most likely, this therapeutic effect was enhanced by preconditioning the patients prior to the transplantation of the gene-modified cells, as preclinical studies have demonstrated that preconditioning might not only facilitate efficient engraftment of gene-transduced cells in the bone marrow but might have also beneficial effects in endogenous microglia turn over (Capotondo et al, 2012). Since the overall outcome was successful and 10–11% gene modified cells already showed therapeutic effects comparable to or even better than those obtained after conventional HSCT, two more patients have been enrolled in this trial (Cartier et al, 2009). Following the same concept, a clinical gene therapy study for MLD, a demyelinating lysosomal storage disorder resulting from arylsulphatase A (ARSA) deficiency was initiated in 2010 in Milan. ARSA overexpression was demonstrated throughout the haematopoietic lineages and in the cerebrospinal fluid resulting in substantial therapeutic effect with no disease progression in any of the eight infantile patients treated (Biffi et al, 2013; Montini et al, 2013).

Treatment of β-haemoglobinopathies, one of the most prevalent group of inherited disorders worldwide, has been in the interest of gene therapy for many years. However, the formidable challenges associated with the temporal and tissue restricted expression of the β-globin gene have delayed the translation of basic research into the clinic. Despite this, gene therapy for β-thalassaemia caused by β-globin deficiency was started in 2007. The SIN-lentiviral vector used in this study contained a mini-globin gene with its introns and the 3′-enhancer region, a minimal version of the β-globin promoter and locus control region and two copies of the 250-bp core element of the cHS4 chromatin insulator. An adult patient was transplanted with *ex vivo* gene modified HSCs and became independent of red blood cell transfusions 1 year later. However, most of the therapeutic benefit was associated with the expansion of a myeloid-restricted cell clone, in which lentiviral vector integration caused the induction of a stable, aberrantly spliced form of the tumour suppressor gene *HMGA2* leading to benign clonal expansion (Cavazzana-Calvo et al, 2010). In a similar study recently opened in the US, a lentiviral vector containing the full-length β-globin gene including its locus control region was used. Two patients have been treated to date, however the observation time post-transplantation is currently too short to make any conclusive statements (Boulard et al, 2013).

With the exception of the β-thalassaemia study mentioned above, one common observation made in all clinical trials in which lentiviral vectors were used, is the high repertoire of clones contributing to gene marked haematopoiesis in bone marrow CD34^+^ cells and peripheral blood myeloid, T and B cells of treated patients. For example, in the WAS trials, high numbers of unique insertions were found in peripheral blood cells in patients over time and no signs of sustained expansion of individual clones were observed so far. In the Italian WAS study, 33,363 unique insertions with different clones contributed to gene-marked haematopoiesis throughout time (last point analysed 18 months after gene therapy; Aiuti et al, 2013). Similarly, integration site analysis of three patients in the MLD trial showed a polyclonal pattern of gene marking up to the last time point analysed (18 months after gene therapy) with no concerning events despite high gene-marking levels *in vivo* in the range between 45 and 80% (Biffi et al, 2013). Lastly, a highly diverse clonal repertoire was observed in the lentiviral X-ALD clinical trial as estimated from the analysis of 21,000 unique integration sites up to 62 months follow-up. Moreover, the detection of common integration sites in myeloid and lymphoid lineages argues for efficient transduction of HSCs or multipotent progenitors (Bartholomae et al, 2013). Although the observation time in most of the above mentioned trials is relatively short, the lack of clonal outgrowth together with the impressive clinical benefits observed in most if not all of the treated patients and the lack of transplantation-related side effects is clear evidence of the power of gene therapy for the treatment of monogenic diseases and may be favoured in the near future for the treatment of patients lacking suitable HSC donors.

In addition to their use in *ex vivo* gene therapy, lentiviral vectors have also been used *in vivo* particularly for the treatment of central nervous system pathologies such as Parkinson's disease and ocular diseases such as the wet form of macular degeneration, Stargardt's disease and Usher syndrome type 1B (http://www.oxfordbiomedica.co.uk/clinical-trials-1/). There are several challenges with the use of lentiviral vectors *in vivo*, for instance the need to manufacture highly-concentrated and highly-purified particles, which could be facilitated by the use of stable producer cell lines (Stewart et al, 2010). In addition, because lentiviral vectors are integrating vectors, it is important to reduce the off-target delivery of these vectors *in vivo*. Novel approaches for cell targeting with engineered lentiviral vector envelope pseudotypes are exciting new developments in this field (Anliker et al, 2010; Frecha et al, 2012; Zhou & Buchholz, 2013).

Of note, lentiviral vectors can be made non-integrating by generating integration-deficient lentiviral vectors (IDLV) resulting in extrachromosomal DNA circles after reverse transcription as (reviewed in (Banasik & McCray, 2009; Mátrai et al, 2010)). This system is highly attractive for gene transfer in post-mitotic tissues as nicely demonstrated by Yañez-Muñoz et al, who used IDLV to introduce the human *RPE65* gene into the retina of a mouse model for Leber congenital amaurosis (Yáñez-Muñoz et al, 2006). However, the levels of expression and transduction efficiency from IDLV vectors are generally low. Nevertheless, IDLV could be very useful in settings where only transient transgene expression is required, as for instance in vaccination approaches or for the delivery of transposases or designer nucleases, which are discussed later in this review (Apolonia et al, 2007; Hu et al, 2010; Lombardo et al, 2007). However, integrase-independent random integration can still occur, but at a comparatively low frequency (<10^−3^; Ebina et al, 2012; Mátrai et al, 2010).

## Alternatives to integrating vectors: AAV-derived vectors

AAV vectors are currently considered as the delivery tool of choice for *in vivo* therapy for treating inherited diseases of post-mitotic tissues. In contrast to lenti- and retroviral vectors, AAV vectors possess a non-enveloped protein capsid and a DNA genome either as single-stranded (native conformation) or as self-complementary DNA (artificial conformation). The latter fold into a double-stranded conformation by intra-molecular base pairing upon being released from the viral capsid leading to a significantly higher level and faster onset of transgene expression compared to vectors delivering single-stranded vector genomes. However, this advantage comes at the price of reducing the coding capacity from approximately 5 to 2.5 kb (McCarty et al, 2003). One of the most interesting features of the parental virus, the replication-deficient, non-pathogenic AAV, is the ability to integrate its genome at a specific site on human chromosome 19 (19q13.3-qter, AAV integration site 1, AAVS1; Büning et al, 2008). The viral packaging signals (inverted terminal repeats, ITRs) flanking the genome and the viral specific non-structural Rep proteins are required for site-specific integration. AAV vectors currently in use are, however, gutless vectors, *i.e*. devoid of all viral open reading frames, and thus remain pre-dominantly in an episomal form. Therefore, AAV is considered as a non-integrating vector system. As a consequence, long-term correction is restricted to post-mitotic tissue, thus explaining the clinical focus on retina (reviewed in (McClements & MacLaren, 2013), central nervous system (Kaplitt et al, 2007; Leone et al, 2012), liver (reviewed in (Mingozzi & High, 2013)), skeletal and cardiac muscle as target tissues (Kratlian & Hajjar, 2012; Tilemann et al, 2012). While initial studies exploited the prototype AAV serotype 2 vector, the portfolio of AAV vectors has recently been expanded to include additional serotypes and even engineered capsids (Mendell et al, 2010; Mingozzi & High, 2013). Despite their episomal nature, AAV vector genomes can be found integrated in the genome of target cells with a frequency of 10^−4^–10^−5^ with no preference for specific genomic loci, although AAV integration site hot-spots with sequence homology to the human mitochondrial DNA genome were recently reported (Kaeppel et al, 2013; Nowrouzi et al, 2012).

The first gene therapy for an inherited eye disease was reported by three independent clinical trials in 2008 in patients with Leber's congenital amaurosis (LCA), an early onset retinal dystrophy (Bainbridge et al, 2008; Hauswirth et al, 2008; Maguire et al, 2008). In these cases, LCA was caused by mutations in retinal pigment epithelium-specific protein 65 kDa (RPE65) gene that encodes a retionoid isomerase (Maguire et al, 2008; McClements & MacLaren, 2013). As isomerase RPE65 is a key factor of the retinol metabolism and hence of the visual cycle. Owing to the need of continuous supply of 11-*cis*-retinal for function and survival, mutations in RPE65 results in dysfunction and degeneration of photoreceptors and thus in loss of vision (Cideciyan et al, 2013). Overexpression of a functional copy of RPE65 following subretinal injection of AAV2 vectors was well-tolerated and led to improvements in vision. The three trials mainly differed in the promoter, *i.e*. expression was either controlled by the cell type specific hRPE65 promoter (Bainbridge et al, 2008) or by a ubiquitously active promoter (Hauswirth et al, 2008; Maguire et al, 2008). The increase in safety by utilizing a cell type specific promoter frequently comes at the prize of a lower expression level and this was suggested to be responsible for the lower efficacy reported by Bainbridge et al compared to the other two studies, as high expression levels appear to be required for vision improvement in the elderly (Bainbridge et al, 2008; McClements & MacLaren, 2013).

A further interesting finding concerns the immune system. In contrast to liver- and muscle-directed gene therapy trials, in which reactivation of memory T-cell responses resulted in loss of vector-modified cells and thus attenuated therapeutic efficacy (Manno et al, 2006; Mendell et al, 2010), subretinal injection of AAV vectors mounted only low humoral immune responses. As all three trials had shown good safety, low immunogenicity, good tolerability and clinical benefit, treatment of the second eye has started and a number of further gene therapy trials for inherited retinal diseases have been launched or are already ongoing (for further details McClements & MacLaren, 2013). In particular for the follow up studies focusing on RPE65-associated LCA, a recent finding by Cideciyan et al is of importance (Cideciyan et al, 2013). Measuring the outer photoreceptor nuclear layer thickness in treated and untreated eyes revealed that although a lasting improvement in vision was achieved by RPE65 overexpression, photoreceptor degeneration continued. Hence, it seems that besides RPE65 overexpression further interventions are required to counteract the two pathological mechanisms, dysfunction and deregulation of photoreceptors, in order to cure RPE65-associated LCA (Cideciyan et al, 2013).

Similarly to the LCA studies, unilateral local injection of AAV2 vectors expressing glutamic acid decarboxylase (GAD) into the subthalamic nucleus of patients with advanced Parkinson's disease led to improvements in clinical scores (Kaplitt et al, 2007; LeWitt et al, 2011). Consequently, a double-blind, sham-surgery controlled, randomized trial with 66 patients was launched. Again, AAV2 vectors encoding GAD were applied into the subthalamic nucleus, however, this time bilaterally. Again, safety and tolerability was proven. Furthermore, compared to the sham-surgery treated group, clinical benefit for AAV2-GAD-treated subjects was reported, including improved motor scores or reductions in measures of overall severity of the disease (LeWitt et al, 2011). A second neurodegenerative disorder, in which gene therapy was shown to be safe and to improve clinical scores, is Canavan disease. Specifically, the aspartocylase gene (ASPA) encoding an enzyme required to degrade *N*-acetyl-aspartate (NAA) was delivered to the brain by AAV2 vectors. In addition to a decrease of NAA concentration in the brain, which approached normal levels, a stabilization of brain atrophy was observed (Leone et al, 2012).

The first clinical trial for haemophilia B employed intra-muscular injections (Kay et al, 2000), a clinically well-established delivery route for conventional therapeutics. A further clear advantage of muscle as target tissue is the lower risk of vector dissemination compared, *e.g*. with liver and the finding that pre-existing anti-AAV humoral immunity, a frequent challenge for AAV-mediated gene therapy, does not block transduction (reviewed in Mingozzi & High, 2013). However, multiple injections are required for delivering the pre-defined vector dose and—based on animal studies—the risk of triggering immune responses towards the transgene products is higher compared with liver (Mays & Wilson, 2011). The latter issue was considered by restricting enrollment to patients with haemophilia B caused by missense mutations. Although a therapeutically relevant level of factor IX was not obtained presumably due to the relatively low secretion efficacy of muscle fibres and/or the vector dose (reviewed in Mingozzi & High, 2013), this study clearly demonstrated safety and applicability, thus paving the way for further muscle-directed gene therapy trials. By changing from AAV2 to AAV1 vectors and by exploiting a natural-occurring gain-of-function mutant of lipoprotein lipase, ^S447X^LPL, a Caucasian variant naturally associated with enhanced removal of lipoprotein particles from the circulation, researchers successfully overcame this caveat for LPLD (Kastelein et al, 2013).

In addition to skeletal muscle, heart muscle has also become a target tissue for gene therapy in light of the increasing incidence of cardiovascular diseases. Although pre-clinical research on developing optimized AAV vectors for the heart is still ongoing (Yang & Xiao, 2013), results of calcium upregulation by percutaneous administration of gene therapy in cardiac disease (CUPID) indicate that AAV1 vectors, the same serotype as for LPLD, successfully and safely transduce human cardiac tissue following antegrade epicardial coronary artery infusion (Jessup et al, 2011). CUPID was launched in the US in 2008 (Kratlian & Hajjar, 2012) to treat patients with advanced heart failure by overexpression of the sarcoplasmic reticulum calcium ATPase pump (SERCA2a). SERCA2a was chosen as a target because expression of this protein, which is essential for calcium homeostasis, is decreased in heart failure leading to elevated end-diastolic calcium (Ca) levels, prolonged Ca re-uptake and a decrease in systolic calcium. In addition to the decreased expression levels, conditions in failing heart negatively impacts on the function of the remaining SERCA2a (reviewed in Kratlian & Hajjar, 2012). AAV1-mediated overexpression of SERCA2a, first assayed within an open-label Phase I trial, demonstrated safety as well as clinical benefit in several of the patients. Based on these results a Phase II trial with 39 patients was designed (Jessup et al, 2011; Tilemann et al, 2012). Patients were randomly assigned to receive either a low, middle or high vector dose or placebo. All vector-treated patients exhibited a decreased frequency of cardiovascular events (Tilemann et al, 2012). In particular, the high-dose group met the pre-specified success criteria, which included decreased heart failure symptoms, improved functional status, left ventricular function/remodelling and clinical outcome (Jessup et al, 2011), again strongly arguing for a clinical benefit of AAV1.SERCA2a in patients with advanced heart failure.

Since insufficient levels of factor IX secretion were obtained when choosing muscle as target tissue, High and colleagues focused subsequently on liver, which possesses a significant greater capacity for secretion of factors to the circulation (Manno et al, 2006) and which is reported to trigger tolerance towards transgene products delivered by AAV vectors (Mays & Wilson, 2011). While therapeutic levels were achieved upon when deliver of AAV2 vectors via the portal vein, factor IX concentration decreased in some of the patients a few weeks after gene therapy. This phenomenon had not been observed in pre-clinical studies and was explained to be due to re-activation of memory T cells recognizing AAV capsid proteins (reviewed in Mingozzi & High, 2013). An alternative explanation suggested by mouse studies is the induction of a cytotoxic T-cell response against an epitope produced from the factor IX transgene upon, *e.g*. usage of an alternative reading frame (Li et al, 2009).

Memory T-cell re-activation may have been caused when a transient, asymptomatic liver inflammation occurred during which AAV-transduced hepatocytes were lost. Prompted by these observations, Nathwani et al changed the vector serotype from AAV2 to AAV8 and employed the self-complementary vector genome conformation (Nathwani et al, 2011). The rationale behind this decision was the assumption that a threshold vector particle dose is required for triggering adaptive anti-capsid immune responses, which can presumably be avoided by using a serotype with lower prevalence in the human population and with a higher transduction efficacy for liver compared to AAV2, and by an improved expression level by changing from single-stranded AAV vector genomes to the self-complementary conformation. An additional, remarkable change to former protocols exploited by this study is the use of peripheral vein infusion as the application route, which appeared to be safe and resulted in a dose-escalation study with clear clinical benefit for the patients. Furthermore, although immune responses were also observed, a short course of glucocorticoids was sufficient to sustain therapeutic efficacy of the gene therapy (Nathwani et al, 2011).

Reviewing AAV-mediated *in vivo* gene therapy reveals a remarkable safety and efficacy record. However, relatively high vector doses are currently required to achieve therapeutic benefit and the broad tropism—a feature shared by all serotypes—imposes the intrinsic risk for off-target transduction. Capsid engineering, *i.e*. modification of the viral capsid, is employed to overcome both of these obstacles and also holds promise for the development of vectors that can be applied in patients with a pre-existing anti-AAV humoral immunity (Büning et al, 2008). As for other viral vector systems, AAV transcriptional as well as post-transcriptional strategies are under development to improve cell type specificity or to restrict transgene expression to a certain developmental stage.

## Beyond vector design: improving gene therapy by targeted gene correction

**Figure 4:**
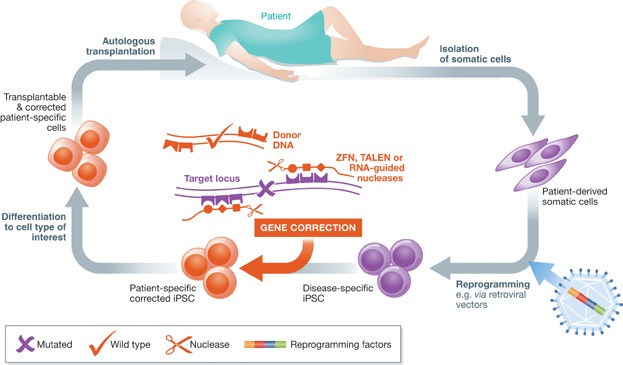
Proposed concept of designer nuclease-mediated correction of patient-specific iPSC for autologous transplantation Patient-specific iPSC can be generated from somatic cells (*e.g*. fibroblasts, blood cells) and reprogrammed into pluripotent stem cells as discussed in the text. Targeted gene correction can be mediated via either zinc-finger nucleases (ZFN), transcription activator like effector nucleases (TALEN) or RNA guided-nucleases of patient-derived cells either before or after reprogramming to iPSC. Disease-corrected somatic cells can be derived from the iPSC and reintroduced into the patient as an autologous transplant.

Despite accumulating data on improved vectors, it should be kept in mind that none of the concepts discussed so far completely alleviates the risk of insertional transformation associated with the use of integrating vectors. To further reduce this risk, approaches aiming at site-directed gene correction are currently under evaluation. Based on the functional dissociation of transcription factors into a DNA-binding domain and a transcription regulatory domain, genetic scissors have been developed by fusing DNA-binding domains to the catalytic domain of endonucleases. These designer nucleases specifically introduce a double-strand DNA break (DSB) at a specific locus recruiting the DNA repair machinery to this site. If an exogenous DNA with homologous arms to the sequence adjacent to the DSB (donor DNA) is provided *in trans*, homologous recombination occurs resulting in the integration of the endogenous sequences at a specific genomic site (Fig 4). If no donor DNA is provided, DSB are corrected by the non-homologous end joining (NHEJ) repair machinery creating mutations/deletions at the DSB site. Zinc-finger nucleases (ZFN), transcription activator like effector nucleases (TALEN) and more recently RNA-guided nucleases (CRISPR/Cas9) have been engineered for this purpose (as reviewed in Mussolino & Cathomen, 2013; Perez-Pinera et al, 2012). The fact that a rationally designed single guide RNA can recruit a corresponding nuclease to virtually any spot of the human genome has interesting perspectives for molecular therapies. This may facilitate the ‘vectorization’ of this strategy and even allow ‘multiplexing’ of several independent interventions as recently demonstrated (Wang et al, 2013). These technologies will allow site-directed integration of a therapeutic cassette into a defined locus in the target cell, thus minimizing dysregulation of gene expression at the integration site, while protecting the therapeutic cassette from epigenetic effects. One of these ‘safe harbour’ integration sites is the AAVS1 locus, which corresponds to the common integration site of AAV, found between exon 1 and intron 1 of the protein *phosphatase 1 regulatory subunit 12C* gene. Zinc-finger endonuclease-mediated site-specific recombination at this locus results in sustained transgene expression with no alterations in the transcriptional pattern of adjacent genes (Lombardo et al, 2011; Sadelain et al, 2012). The translation of this technology into the clinics was initially delayed by suboptimal specificity, resulting in off-target genotoxicity and cytotoxicity of the designer nucleases. However, new generations of zinc-finger nucleases and TALEN have a significantly improved safety profile (Gabriel et al, 2011; Mussolino et al, 2011). A multicentre clinical trial is ongoing that aims at specific gene disruption by a zinc-finger nuclease targeting the HIV-1 co-receptor CCR5 to protect T cells from new infection (Burnett et al, 2012; Perez et al, 2008). More than 30 HIV-patients have been treated with zinc-finger nuclease-modified T cells containing a disrupted CCR5 locus, resulting in a sustained increased in CD4 counts, most likely resulting from long-term maintenance of CCR5-modified central memory CD4 cells (Lee et al, 2013). In the future, site-directed integration might eventually substitute for (semi-) randomly integrating vectors, provided comparable gene delivery and recombination efficiencies can be achieved.

## The perspectives of induced pluripotent stem cells in gene and cell therapy

Pluripotent stem cells (PSC) can differentiate into cells of all three germ layers, including ectoderm (*e.g*. neurons, epidermis), mesoderm (*e.g*. blood, cardiac cells, muscle) and endoderm (*e.g*. pancreas, liver). Given that PSC can be generated from any individual and as such in a patient-/disease-specific manner from adult somatic cells, they create intriguing options for disease modelling, drug testing, developmental studies and combined gene and cell therapy.

To understand the underlying networks governing reprogramming fate decisions in this rapidly growing area, a number of important studies are briefly mentioned here. The first formal proof that mature cells can be ‘reprogrammed’ into immature PSC was obtained in1962 by John Gurdon and coworkers, who transferred the nucleus from a mature intestinal cell to replace the immature cell nucleus in an egg cell of a frog. This ‘genetically’ modified egg subsequently developed into a normal *Xenopus* tadpole (Gurdon, 1962). Employing a similar nuclear transfer technique, Ian Wilmut reported in 1997 the birth of the sheep Dolly, the first clone produced from a somatic cell taken from an adult mammal (Wilmut et al, 1997). Of note, Tachibana et al demonstrated recently that human embryonic stem cells (ESC) could be derived by somatic cell nuclear transfer (Tachibana et al, 2013). Taken together, these studies provide clear evidence that the cytoplasm of oocytes reprogrammed the transplanted somatic cell nuclei to pluripotency and that these PSC could be differentiated into a variety of cell types of all three germ layers, and in the case of Dolly, into a living individual.

In 2006, Shinya Yamanaka discovered the pluripotency factors (*i.e*. Oct4, Sox2, Klf4, c-Myc), which were sufficient and necessary to reprogramme mature somatic cells into so-called *induced* pluripotent stem cells (iPSC; Takahashi & Yamanaka, 2006). This seminal finding set the stage to generate disease-specific human iPSC from patients' somatic cells (Park et al, 2008; Yu et al, 2007). Since then, a steadily growing list of patient-specific iPSC resembling genetic diseases with either Mendelian or complex inheritance has been generated (Onder & Daley, 2012). These cells create an important reservoir for further research to elucidate disease pathologies and to develop new therapeutic options. This is especially critical in rare diseases, where patient material is severely limited.

Patient-specific iPSC can be differentiated into key cell types to identify underlying disease-associated phenotypes and pathologies during development and in the differentiated mature progeny. These cells serve as discovery and screening platforms to identify common signalling pathways and to discover small molecule drugs and molecular therapies that potentially reverse the disease phenotype.

Gene therapy represents a significantly powerful therapeutic option to repair the known underlying genetic causes in monogenetic disease-specific iPSC. As for the generation of iPSC, many opportunities exist for genetic correction of iPSC, including integrating and non-integrating viral vectors, non-viral vectors (*e.g*. Sleeping Beauty transposon), episomal, mRNA and protein delivery. Retroviral vectors are in particular interesting tools for the genetic modification of iPSC, since the introduced therapeutic expression cassette is stably integrated into the iPSC's genome and transmitted to all differentiated progeny. Thus, ideally one carefully conducted treatment should allow the permanent correction/alleviation of disease symptoms. Moreover, the monoclonal nature of iPSC and the screening of potential off-target effects by whole genome sequencing allow for the identification of genetically corrected iPSC meeting all safety requirements. Also, the risks of insertional transformation (see above) can be strongly decreased by screening for ‘safe-harbour’ integrations. Using this strategy, Papapetrou et al demonstrated that ∼10% of integrations of a lentivirally encoded transgene occur in safe harbours and permitted sustained globin expression in β-thalassaemia iPSC and their differentiated progeny (Papapetrou et al, 2011). Similarly, correction of CGD by targeted integration of a therapeutic cassette into the AAVS1 locus was shown to restore superoxide production in granulocytes derived from the targeted iPSC (Zou et al, 2011). Targeted correction of the disease-causing mutations by homologous recombination in iPSC is well within reach and has been demonstrated for various disease-specific iPSC (Nakayama, 2010; Zou et al, 2011).

The growing number of studies combining approaches for gene and cell therapy underlines the potential of iPSC derived strategies in disease modelling and therapeutic options. However, improved and more reliable differentiation protocols leading to transplantable cell types that integrate into their natural niches *in vivo*, *e.g*. engraftable HSC, will have to be developed (Suzuki et al, 2013). This may ultimately lead to autologous transplants, which are compatible to the recipient's immune system (Fig 4; Araki et al, 2013a, 2013b). Interestingly, a first clinical trial using iPSC—to be conducted in Japan—is already in sight. In this study, Masayo Takahashi, ophthalmologist at the RIKEN Center for Developmental Biology in Kobe, plans to use iPSC-derived cells for the treatment of the debilitating eye disease age-related macular degeneration (http://blogs.nature.com/news/2013/02/embryo-like-stem-cells-enter-first-human-trial.html http://www.nature.com/news/stem-cells-cruise-to-clinic-1.12511).

In summary, combined gene and cell therapy using iPSC may expand our reservoir of molecular therapies and may offer interesting perspectives for the treatment of various disorders.

**Pending issues**

Long-term efficacy and safety of gene therapeutic drugs

Improved benefit/risk ratio

Commercialization

Risk assessment during transplantation

AAV immunogenicity

Target cell selective gene transfer

Translation of iPSC technology into the clinics

iPSC differentiation into transplantable cells

## Conclusion

Gene therapy is on its way to fulfill the early promises made just two decades ago despite the occurrence of severe side effects observed in the initial clinical trials. The risks associated with gene therapy are already being successfully addressed or are the focus of current research as presented here. Multicentre studies and gene therapy consortia are currently favoured not only in the view of sharing resources but more importantly, to obtain more informative and reliable data by increasing patient numbers included in the Phase I/II clinical trials. The very recent approvals of gene therapeutic agents for the European market are a milestone in the field of gene therapy and raise hope for many patients suffering from orphan diseases as well as many other more common illnesses. These facts reflect the growing acceptance that gene therapy might no longer be considered only as an alternative therapy for terminally sick patients who failed conventional treatment, but could also become the first-line treatment for a wide variety of diseases in the near future. The recent therapeutic successes observed in many treated patients have encouraged pharmaceutical companies to support the development of gene therapy including Phase I/II clinical trials (Mavilio, 2012) (http://www.uphs.upenn.edu/news/News_Releases/2012/08/novartis/). Their involvement will certainly boost the transition from bench to bedside for the benefit of the patients.

## For more information

Database of Clinical Trials

http://www.clinicaltrials.gov/

Gene Therapy Trials Worldwide (Overview)

http://www.wiley.com//legacy/wileychi/genmed/clinical

Gene and Cell Therapy Societies

http://www.genetherapynet.com/societies.html

Gene and Cell Therapy Terminology

http://www.asgct.org/about_gene_therapy/terminology.php

Online Catalog of Human Genes and Genetic Disorders

http://www.omim.org/

European Medicines Agency

http://www.ema.europa.eu

U.S. Food and Drug Administration—Cellular & Gene Therapy Products

http://www.fda.gov/biologicsbloodvaccines/cellulargenetherapyproducts/default.htm
